# Comparative effectiveness and safety of oral anticoagulants for atrial fibrillation in real-world practice: a population-based cohort study protocol

**DOI:** 10.1136/bmjopen-2016-013263

**Published:** 2016-11-24

**Authors:** Anne Holbrook, Colin Dormuth, Richard Morrow, Agnes Lee, Sue Troyan, Guowei Li, Eleanor Pullenyegum

**Affiliations:** 1 Division of Clinical Pharmacology & Toxicology, McMaster University, Hamilton, Ontario, Canada; 2 Department of Clinical Pharmacology & Toxicology, St Joseph's Healthcare Hamilton, Hamilton, Ontario, Canada; 3 Department of Clinical Epidemiology and Biostatistics, McMaster University, Hamilton, Ontario, Canada; 4 Department of Anesthesiology, Pharmacology and Therapeutics, University of British Columbia, Vancouver, British Columbia, Canada; 5 Faculty of Medicine, University of British Columbia, Vancouver, British Columbia, Canada; 6 Child Health Evaluative Sciences, Hospital for Sick Children, Toronto, Ontario, Canada

**Keywords:** VASCULAR MEDICINE

## Abstract

**Introduction:**

Anticoagulants are arguably the most important drug family of all, based on the frequency and duration of their use, and the clinical importance and frequency of benefits and harms. Several direct acting oral anticoagulants (DOACs) have recently joined warfarin for the treatment of atrial fibrillation, with a resultant significant expansion in use of oral anticoagulants (OACs). Our objectives are to compare safety and effectiveness of DOACs versus warfarin in a full population where anticoagulation management is good and to identify which types of patients do better with DOACs versus warfarin and vice versa.

**Methods and analysis:**

This is a retrospective cohort study of all adults living in British Columbia who have a diagnosis of atrial fibrillation in hospital or medical service data, and a first prescription for an OAC. Coprimary outcomes are ischaemic stroke and systemic embolism (benefit) and major bleeding (harm). Secondary outcomes include net clinical benefit (composite of stroke, systemic embolism, major bleeds, myocardial infarction, pulmonary embolism and death), drug discontinuation and individual composite item occurrence. We will estimate the effects of treatment in a 2-year follow-up period, using time-to-event models with propensity score adjustment to control confounding. Secondary analyses will examine ‘as treated’ outcomes.

**Ethics and dissemination:**

The protocol, data creation plan, privacy impact statement and data sharing agreements have been approved. Dissemination is planned via conferences and publications as well as directly to drug policy leaders. Information on the overall comparative effectiveness and safety of DOACs versus warfarin in a country with high quality anticoagulation management, as well as for vulnerable subgroups, will be an important addition to the literature.

**Strength and limitations of this study**

- This population-based retrospective cohort study minimises patient selection bias and prescribing channelling bias while examining comparative effectiveness and safety of oral anticoagulants.
- By linking patient-specific laboratory data, we hope to add additional essential information to determine predictors of outcomes.
- Although this is a large study of real-world patients, confounders, particularly unmeasured confounders, may create bias.
- The process of matching for purposes of comparative analysis leaves observational studies vulnerable to the loss of eligible patients.

## Introduction

Comparative effectiveness research (CER) priorities of the Institute of Medicine have highlighted the treatment of atrial fibrillation and the comparative effectiveness of anticoagulants as priority topics.^1^ This is because of the widespread use of oral anticoagulants (OAC), particularly in elderly populations, their major benefit in preventing morbid and fatal thrombotic events and their potential for major harm, which is largely bleeding. Large rigorous randomised trials have been critical to allowing direct acting oral anticoagulants (DOACs) on to the market, but have not settled whether their rapid uptake in clinical practice, probable use in patient groups beyond those included in the trials, and use in countries where international normalised ratio (INR) monitoring is relatively high quality generates similar benefits and harms compared to warfarin.^2–5^

Currently more than 7 million prescriptions are dispensed annually for OACs in Canada.^6^ Warfarin is one of the most cost-effective medications in current use, with a 68% reduction in stroke rates and a significant decrease in all-cause mortality in atrial fibrillation, at a negligible drug cost.^7^ Warfarin's narrow therapeutic index, the variability of its effect in some patients and concern regarding drug and food interactions mandate laboratory INR monitoring of its anticoagulant effect.^7^ The need for monitoring and dose adjustment is reassuring to some patients and is the ‘gold standard’ adherence check for drugs, but burdensome for other patients and for physicians. The DOACs are given in fixed doses and do not require (or benefit from) INR monitoring. Three of these, dabigatran, rivaroxaban and apixaban, are now available in Canada. Within a year of dabigatran launch in 2010, worldwide sales were estimated at US$1 billion annually.^8^ However, by 2011 the Institute for Safe Medication Practices (ISMP) and the Food and Drug Administration (FDA) noted that dabigatran was the leading cause of drug-related serious harm and death reported to the FDA in 2011.^9^ ^10^

Several critical issues, outlined below, may significantly influence the relative effectiveness and safety of DOACs compared to warfarin in real-world practice and potentially produce different results than the pivotal trials. Patient characteristics, several of which have not previously been available to study, may predict a better benefit:harm ratio with warfarin versus DOACs or vice versa. Analyses of these features will improve the appropriate tailoring of anticoagulant therapy to individual patients.

The absolute (as opposed to relative) differences between DOACs and warfarin are quite small with 95% CIs ranging from 2 more to 8 fewer events per 1000 patients for stroke or systemic embolism, and 11 fewer to 6 more per 1000 for major bleeding.^11^ Consequently, there are several reasons why the advantages of DOACs might not be realised in usual clinical practice:

1. DOACs, especially dabigatran, are contraindicated in patients with severe renal impairment because of accumulating drug concentrations.^12^ Atrial fibrillation is primarily a disease of the elderly, older patients are vulnerable to rapid decline in renal function, and patients with renal impairment are at significantly higher risk of bleeding even without anticoagulation.
2. DOACs have no marker (as the INR is for warfarin) for their effectiveness, safety or adherence. In addition, DOACs may produce more adverse symptoms (eg, dabigatran and gastrointestinal symptoms) than warfarin leading to undetected no-adherence. Finally, all DOACs have relatively short half-lives, and missed doses may have more impact on anticoagulation outcomes. While patient adherence is closely monitored in phase III clinical trials, monitoring in usual care is much more variable.
3. Although antidotes are becoming available, their absence left no reliable way to stop DOAC-associated bleeding, and the antidotes will be very expensive and restricted to hospital settings.^13^ Patient monitoring and follow-up is less vigilant in clinical practice compared to clinical trials, thus the outcomes of bleeding may not be as favourable in usual practice.
4. There are several DOACs available, each with separate dosages and dosing schedules depending on patient age and risk factors. Doses for atrial fibrillation differ from those for other indications. This will create confusion for prescribers, the vast majority of whom do not specialise in this area, and may lead to errors. Similarly, information on switching anticoagulants, bridging with DOACs, follow-up intervals, DOAC drug interactions, etc, is sparse.
5. Time in therapeutic range (TTR) for INR is a good surrogate for warfarin safety and effectiveness.^14^ Study centres including Canadian centres, which maintained good average TTRs, saw no significant advantage to DOACs in clinical outcomes.^15^ It is unclear whether routine clinical practice maintains a good average TTR.

In summary, although the relative benefit:harm ratio of the two comparators in usual clinical practice remains uncertain, the importance of the outcomes is universal and unequivocal. Stroke, systemic arterial emboli and pulmonary emboli are frequently devastating and can be fatal, and major bleeding carries a death rate of 10% overall, 40% if intracranial.^16^ Cost-effectiveness and budget impact are also ongoing issues, as DOACs are much more expensive than warfarin. With the major clinical concerns in mind plus the rapidly increasing use of DOACs, there is an urgent and unmet need to evaluate outcomes in real clinical practice.

Observational studies using population-based health databases are increasingly used for CER because they can rapidly analyse large sample sizes with relevant outcome data across multiple comparators, entirely based in actual clinical practice and without any ethical concern about deliberate exposure to harm.^2^ ^17^ Their main disadvantage relates to bias associated with non-random allocation, a bias now reduced with innovation in methods of case selection, follow-up, analysis and adjustment.^18–20^ Another essential component of comparative effectiveness and safety is the determination of the patient characteristics that predict a superior benefit:harm profile with one drug versus the other.

The linked healthcare databases of British Columbia (BC), Canada, are unusual internationally, as they include the entire population (∼4.7 million people). These databases include medications dispensed at community pharmacies (excluding the 4% who are federally insured), hospitalisations, medical services and vital statistics for BC residents.^21^ In addition, this study will incorporate a novel patient-level laboratory link for those laboratory results that are required to explain the comparative effectiveness and safety of anticoagulants. Previous studies comparing DOACs individually or as a group versus warfarin have either suffered from a lack of population-level data coverage (producing biased results) or examine only one outcome (not comprehensive).^22–27^

Our primary objectives for this study are as follows: (1) to clarify the overall comparative effectiveness and safety of DOACs versus warfarin in clinical practice where high quality is expected, (2) to compare and contrast our results with the relevant phase III randomised trials and (3) to identify the key predictors of superior effectiveness and safety (which drug is better for which patients) in vulnerable populations—predictors which are very likely to include INR control quality in the warfarin patients, remote location (probable lack of ready access to INR testing), access to certain specialists and presence of severe renal impairment.

## Methods and analysis

### Study design

The study will use a retrospective cohort design in which the treatment effects of new use of DOACs compared to warfarin are estimated, adjusted by propensity scores, for residents with a diagnosis of atrial fibrillation. This will allow us to preserve the population-based longitudinal data collection advantages, minimise selection and confounding biases and provide outcome data amenable to rapid clinical interpretation (eg, time to event analysis, relative risks, absolute risks, numbers needed to treat (NNT)).

### Study cohort

The source population includes all BC residents aged 18 years or older, an estimated 3.6 million individuals. Our access to BC data includes de-identified data extracts from PharmaNet, Medical Services Plan billings (physician payments), the hospital Discharge Abstract Database (DAD), BC Vital Statistics death records and LifeLabs laboratory data. All data are accessed once appropriate approvals and payment are received, using Population Data BC secure research servers.^21^ Our sampling frame will be BC residents enrolled with the Medical Services Plan during the 12 months before starting an anticoagulant drug (index prescription). Eligibility for inclusion in the cohort will be those with a diagnosis of atrial fibrillation in hospital or medical services data within the 36 months prior to the index prescription.

**Figure 1:**
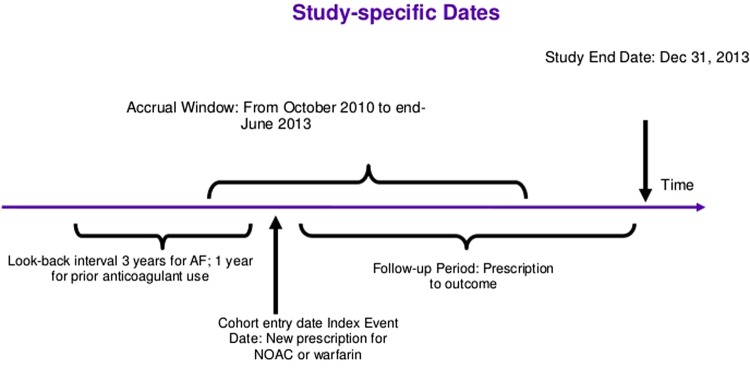
Time frame definitions.

Anticoagulant exposure will be determined from dispensed prescription database records, beginning 1 October 2010 and continuing until study end (details in figure 1). New users of warfarin, dabigatran, rivaroxaban and apixaban will be identified. To identify new users, a look-back observation period of 12 months prior to the index prescription date will be used and must indicate no anticoagulant use during this period. The index date will be defined as date of the new prescription for OAC. Determination of exposure, as far as possible, will be blinded to patient outcome.

### Outcomes

The coprimary outcomes, chosen for their clinical importance and their similarity to those in the pivotal trials, will be ischaemic stroke and systemic embolism, and major bleeding defined as bleeding requiring hospitalisation. Intracranial haemorrhage (ICH) will be counted as part of major bleeding. Secondary outcomes include: (1) net clinical benefit, defined as a composite of ischaemic stroke, systemic embolism, major bleeds, myocardial infarction, pulmonary embolism and death. This composite was used in RE-LY and is a good summary representation of clinically important benefit and harm. All of these outcomes will be identified in hospital DAD, or vital statistics data, and have been validated;^28–32^ (2) discontinuation using dispensing gap analysis. Discontinuation is defined as a calculated gap of more than 30 days in therapy and (3) the individual clinical outcomes that are part of net clinical benefit composite plus components of major bleeding (notably ICH and gastrointestinal bleeding). By incorporating all relevant serious events into the net clinical benefit, we avoid the problem of competing risks.^33^

### Data codes

Data codes are available in online supplementary appendix 1 (available at our website https://rsjh.ca/holbrook/CES-AC_Protocol_Appendices_Jun29_16.pdf).

### Follow-up

Outcomes will be counted in a follow-up window to 24 months postindex prescription, death, exit from BC or the end of the study window. We will censor follow-up for death or departure from the province, but not clinical outcomes, in order to gather information on the clustering of important clinical events (eg, patient has a bleed, discontinues warfarin, then has a vascular event and dies).

### Analysis plan

The primary analysis will be intention-to-treat, meaning that if the index prescription is for warfarin, then any outcome events are attributed to warfarin whether switching occurs or not. This mimics the conservative, recommended analysis in clinical trials. However, this approach may underestimate benefit and harm, therefore a secondary ‘as-treated’ analysis will explore outcomes based on actual treatment during follow-up. Although both analyses occur after baseline confounders are adjusted for, the as-treated analysis requires further adjustments for measured time-varying confounding and selection bias using inverse-probability weighting.^34^ Switching between anticoagulants will be further explored by examining comparative duration on each anticoagulant as well as switch frequency.

For the main analysis, the effects of treatment with the use of DOACs versus warfarin will be estimated from time-to-event models (Cox proportional hazards or Poisson). Risk modifying effects such as age, sex, dose and risk factors (including congestive heart failure, hypertension, diabetes, prior stroke, prior major bleed, renal or liver failure, interacting concomitant medications, alcohol abuse) will be evaluated using tests of interaction. Tests of interaction will be used to identify key subgroups in which warfarin may be better than DOACs or DOACs better than warfarin, using the two coprimary outcomes and net clinical benefit as defined above. Models can simultaneously control for potential confounders such as cardiovascular disease, rural/remote location of residence, physician practice—all estimated at index date (further details in online supplementary appendix 1). Sample size is unlikely to allow individual DOAC versus DOAC comparison, but outcomes will be tabulated by individual drug.

Community-based outcome event rates and the RE-LY trial suggest a coprimary outcome event rate of ∼12% per year for warfarin versus 10% per year for DOACs.^3^ ^35^ Assuming conservatively that accrual extends over 3 years and that patients have a minimum of 1 year of follow-up, and that 67% of the sample is on warfarin, an analysis based on outcomes at 1 year would require 6000 patients on warfarin and 3000 patients on DOACs for 80% power. Since our analysis will be time-to-event rather than using binary outcomes at 1 year, we expect to have greater power than estimated.

Missing data, including missing laboratory results, are represented as variables in the propensity score. All analyses will be carried out using SAS. 95% CIs for the estimates will be calculated, with two-tailed, p<0.05 considered significant. Absolute risk difference and NNT to benefit or harm will be calculated. Analysis and reporting will be compliant with Strengthening the Reporting of Observational Studies in Epidemiology (STROBE) recommendations.^36^

### Control for confounding

New users of DOACs are likely to differ in several characteristics from new users of warfarin. We expect them to be younger and with less comorbidity. To control for confounding, we will adjust estimates by age, sex and propensity score. Propensity scores will be computed by multiple logistic regression of the logit of the conditional probability of allocation to warfarin versus any DOAC (conditional on patient being a new user of either anticoagulant) regressed on potential confounders at baseline. Among the variables considered will be sex, dose, CHADS_2_ (Congestive heart failure, hypertension, age, diabetes, stroke - 2 points) score, and HAS-BLED score (hypertension, abnormal renal or hepatic function, previous stroke, bleeding, labile INR, age, drugs/alcohol), other vascular events, concomitant medications, drug plan coverage, remote location, neighbourhood income quintile, physician specialty, haemoglobin and year of cohort entry. We plan to evaluate conventional and high-dimensional propensity scores, the latter estimated using preselected and empirically chosen variables from medication, hospital and medical services data.^37^

Analyses of switching patterns, including the duration of OAC exposure and the switching frequency, will be treated as time-dependent confounders and controlled by marginal structural modelling.^38^

## Ethics and dissemination

Required ethics approvals from Hamilton Integrated Research Ethics Board Application #16-643-C and UBC Clinical Research Ethics Board Application #H13-00868, and data sharing agreements with PopData-BC and LifeLabs Medical Laboratory Services, have been obtained prior to this study. Secure access and storage of data and data linkage are governed by Population Data BC. The necessary data access request templates, privacy impact analyses and research service agreements are complete.

Results will be published in a peer-reviewed journal electronically and in print. They will also be disseminated to the national drug plan managers and to the national drug safety and effectiveness leaders. The study results will be an important addition to the literature and for policymakers for four main reasons: (1) the usage of the DOACs has increased sharply since their launch adding more than US$100 million annually to public plan drug budgets in Canada, (2) their comparative efficacy and safety in pivotal trials was near identical for countries with high-quality warfarin management thus bringing cost-effectiveness into question, (3) the OACs overall are a very high benefit/high harm class of drugs and (4) our study using universal population coverage data will avoid biases present in other real-world studies.

## References

[1] Institute of Medicine (IOM). Initial national priorities for comparative effectiveness research. Washington (DC): The National Academies Press, 2009 http://www.nap.edu/openbook.php?record_id=12648

[2] Chokshi DA, Avorn J, Kesselheim AS Designing comparative effectiveness research on prescription drugs: lessons from the clinical trial literature. Health Aff (Millwood) 2010;29:1842–8. 10.1377/hlthaff.2010.0843 20921484

[3] Eikelboom J, Parekh A, Pogue J Dabigatran versus warfarin in patients with atrial fibrillation. N Engl J Med 2009;361:1139–51. 19717844 10.1056/NEJMoa0905561

[4] Patel MR, Mahaffey KW, Garg J Rivaroxaban versus warfarin in nonvalvular atrial fibrillation. N Engl J Med 2011;365:883–91. 10.1056/NEJMoa1009638 21830957

[5] Granger CB, Alexander JH, McMurray JJV Apixaban versus warfarin in patients with atrial fibrillation. N Engl J Med 2011;365:981–92. 10.1056/NEJMoa1107039 21870978

[6] Weitz JI, Semchuk W, Turpie AGG Trends in prescribing oral anticoagulants in Canada, 2008–2014. Clin Ther 2015;37:2506–14.e4. 10.1016/j.clinthera.2015.09.008 26481493

[7] You JJ, Singer DE, Howard PA Antithrombotic therapy for atrial fibrillation: antithrombotic therapy and prevention of thrombosis, 9th ed: American College of Chest Physicians Evidence-based Clinical Practice Guidelines. Chest 2012;141(2 Suppl):e531S–e75S. 10.1378/chest.11-2304 22315271 PMC3278056

[8] Allyn B Blood-thinner Pradaxa target of mass-claims suit. *USA Today*, August 19 2012 http://www.usatoday.com/news/health/story/2012-08-19/pradaxa-blood-thinnerlawsuit/57134628/1?csp=34news

[9] Institute for Safe Medication Practices. QuarterWatch: Monitoring FDA MedWatch Reports. Anticoagulants the Leading Reported Drug Risk in 2011 31 May 2012 http://www.ismp.org/quarterwatch/pdfs/2011Q4.pdf

[10] Adverse Event Reporting System (AERS). Food and Drug Administration 2011 (accessed 20 Aug 2012). http://www.fda.gov/Drugs/GuidanceComplianceRegulatoryInformation/Surveillance/AdverseDrugEffects/ucm082193.htm

[11] Wells G, Coyle D, Cameron C Safety, effectiveness, and cost-effectiveness of new oral anticoagulants compared with warfarin in preventing stroke and other cardiovascular events in patients with atrial fibrillation. Canadian Agency for Drugs and Technologies in Health, 2012. 24279001

[12] Boehringer Ingelheim. Advisory committee briefing document 2010:1–168. http://www.fda.gov/downloads/advisorycommittees/…/drugs/…/ucm226009.pdf (accessed 21 May 2011).

[13] Sarich TC, Seltzer JH, Berkowitz SD Novel oral anticoagulants and reversal agents: considerations for clinical development. Am Heart J 2015;169:751–7. 10.1016/j.ahj.2015.03.010 26027611

[14] Holbrook A, Schulman S, Witt DM Evidence-based management of anticoagulant therapy. Antithrombotic therapy and prevention of thrombosis, 9th ed: American College of Chest Physicians Evidence-based Clinical Practice Guidelines. Chest 2012;141(2 Suppl):e152S–e84S. 22315259 10.1378/chest.11-2295 PMC3278055

[15] Gomez-Outes A, Terleira-Fernandez AI, Calvo-Rojas G Dabigatran, rivaroxaban, or apixaban versus warfarin in patients with nonvalvular atrial fibrillation: a systematic review and meta-analysis of subgroups. Thrombosis 2013;2013:640723 10.1155/2013/640723 24455237 PMC3885278

[16] Appelboam R, Thomas EO Warfarin and intracranial haemorrhage. Blood Rev 2009;23:1–9. 10.1016/j.blre.2008.05.001 18583002

[17] Mello MM, Goodman SN, Faden RR Ethical considerations in studying drug safety—the Institute of Medicine report. N Engl J Med 2012;367:959–64. 10.1056/NEJMhle1207160 22913661

[18] D'Agostino RB Jr, D'Agostino RB Sr Estimating treatment effects using observational data. J Am Med Assoc 2007;297:314–16. 10.1001/jama.297.3.314 17227985

[19] GRACE Initiative. GRACE principles. Good research for comparative effectiveness observed. 10 April 2010. http://www.graceprinciples.org (accessed 25 May 2011).

[20] Austin PC, Laupacis A A tutorial on methods to estimating clinically and policy-meaningful measures of treatment effects in prospective observational studies: a review. Int J Biostat 2011;7:6 10.2202/1557-4679.1285 22848188 PMC3404554

[21] https://www.popdata.bc.ca/home (accessed 16 May 2016).

[22] Maura G, Blotiere PO, Bouillon K Comparison of the short-term risk of bleeding and arterial thromboembolic events in nonvalvular atrial fibrillation patients newly treated with dabigatran or rivaroxaban versus vitamin K antagonists: a French nationwide propensity-matched cohort study. Circulation 2015;132:1252–60. 10.1161/CIRCULATIONAHA.115.015710 26199338 PMC4885525

[23] Lauffenburger JC, Farley JF, Gehi AK Effectiveness and safety of dabigatran and warfarin in real-world US patients with non-valvular atrial fibrillation: a retrospective cohort study. J Am Heart Assoc 2015;4:pii: e001798 10.1161/JAHA.115.001798 PMC4579955 25862791

[24] Larsen TB, Rasmussen LH, Gorst-Rasmussen A Dabigatran and warfarin for secondary prevention of stroke in atrial fibrillation patients: a nationwide cohort study. Am J Med 2014;127:1172–8.e5. 10.1016/j.amjmed.2014.07.023 25193361

[25] Laliberte F, Cloutier M, Crivera C Effects of rivaroxaban versus warfarin on hospitalization days and other health care resource utilization in patients with nonvalvular atrial fibrillation: an observational study from a cohort of matched users. Clin Ther 2015;37:554–62. 10.1016/j.clinthera.2015.02.001 25749196

[26] Abraham NS, Singh S, Alexander GC Comparative risk of gastrointestinal bleeding with dabigatran, rivaroxaban, and warfarin: population based cohort study. BMJ 2015;350:h1857. 25910928 10.1136/bmj.h1857 PMC4413863

[27] Larsen TB, Rasmussen LH, Gorst-Rasmussen A Myocardial ischemic events in ‘Real world’ patients with atrial fibrillation treated with dabigatran or warfarin. Am J Med 2014;127:329–36.e4. 10.1016/j.amjmed.2013.12.005 24361757

[28] Raiford DS, Gutthann SP, Garcia Rodriguez LA Positive predictive value of ICD-9 codes in the identification of cases of complicated peptic ulcer disease in the Saskatchewan Hospital automated database. Epidemiology 1996;7:101–4. 10.1097/00001648-199601000-00018 8664388

[29] Arnason T, Wells PS, van Walraven C Accuracy of coding for possible warfarin complications in hospital discharge abstracts. Thromb Res 2006;118:253–62. 10.1016/j.thromres.2005.06.015 16081144

[30] Juurlink D, Preyra C, Croxford R ICES investigative report—Canadian Institute for Health Information Discharge Abstract Database: a validation study. Toronto: Institute for Clinical Evaluative Sciences, 2006 http://www.ices.on.ca/file/cihi_dad_reabstractors_study.pdf

[31] Levy AR, O'Brien BJ, Sellors C Coding accuracy of administrative drug claims in the Ontario Drug Benefit database. Can J Clin Pharmacol 2003;10:67–71. 12879144

[32] Quan H, Li B, Saunders LD Assessing validity of ICD-9-CM and ICD-10 administrative data in recording clinical conditions in a unique dually coded database. Health Serv Res 2008;43:1424–41. 10.1111/j.1475-6773.2007.00822.x 18756617 PMC2517283

[33] Varadhan R, Weiss CO, Segal JB Evaluating health outcomes in the presence of competing risks: a review of statistical methods and clinical applications. Med Care 2010;48(6 Suppl):S96–S105. 10.1097/MLR.0b013e3181d99107 20473207

[34] Hernan MA, Hernandez-Diaz S Beyond the intention-to-treat in comparative effectiveness research. Clin Trials 2012;9:48–55. 10.1177/1740774511420743 21948059 PMC3731071

[35] Rose AJ, Ozonoff A, Henault LE Warfarin for atrial fibrillation in community-based practise. J Thromb Haemost 2008;6:1647–54. 10.1111/j.1538-7836.2008.03075.x 18853483

[36] Von Elm E, Altman DG, Egger M The Strengthening the Reporting of Observational Studies in Epidemiology (STROBE) statement: guidelines for reporting observational studies. Int J Surg 2014;12:1495–9. 10.1016/j.ijsu.2014.07.013 25046131

[37] Schneeweiss S, Rassen JA, Glynn RJ High-dimensional propensity score adjustment in studies of treatment effects using health care claims data. Epidemiology 2009;20: 512–22. 10.1097/EDE.0b013e3181a663cc 19487948 PMC3077219

[38] Robins JM, Hernan MA, Brumback B Marginal structural models and causal inference in epidemiology. Epidemiology 2000;11:550–60. 10.1097/00001648-200009000-00011 10955408

